# Preparation and application of chikungunya pseudovirus containing double reporter genes

**DOI:** 10.1038/s41598-022-13230-0

**Published:** 2022-06-14

**Authors:** Chunyan Su, Kaiyun Ding, Jingwen Xu, Jianchao Wu, Jiansheng Liu, Jiayuan Shen, Hongning Zhou, Hongqi Liu

**Affiliations:** 1 Institute of Medical Biology, Chinese Academy of Medical Sciences and Peking Union Medical College, Kunming, 650118 China; 2 Yunnan Provincial Key Laboratory of Vector-Borne Diseases Control and Research, Yunnan Institute of Parasitic Diseases, Simao Pu’ er, 665000 Yunnan China

**Keywords:** Immunology, Microbiology, Molecular biology

## Abstract

Chikungunya virus (CHIKV), a highly infectious and rapidly spread viral pathogen, is classified as a pathogenic agent at the biosafety level 3. Operation of live authentic CHIKV needs a specific laboratory with the P3 or above containment, which greatly confines the CHIKV-associated studies. To establish an evaluation system of CHIKV that can be utilized in a BSL2 laboratory, we constructed a pseudovirus (PsV) system of CHIKV containing double reporter genes (*ZsGreen1* and *luciferase*). The fluorescent ZsGreen1 is a convenient and cheap reporter for monitoring the efficiency of transfection and titration of PsV. The enzyme luciferase is a sensitive reporter for the application of PsV to neutralization assay or drug screening. The CHIKV PsV produced in this study, with a titer of up to 3.16 × 10^6^ TU/ml, was confirmed by Western blotting and transmission electronic microscopy (TEM). Finally, we developed a microneutralization assay with the CHIKV PsV produced in this study, which was successfully applied to evaluate neutralizing activities of convalescent sera from CHIKV-infected patients. In summary, we have established a convenient and sensitive double-reporter CHIKV pseudovirus system, which provides a safe and effective platform for screening anti-CHIKV drugs and evaluating vaccines against CHIKV.

## Introduction

Chikungunya, also called chikungunya fever (CHIKF), is an acute febrile complications of red rash, muscle pain, joint pain and even central nervous system diseases, which is caused by the arthropod-borne chikungunya viruses (CHIKV)^1^. Outbreaks of CHIKF have been reported in more than 100 countries all over the world, leading to about 1 million cases annually^2^. However, no effective vaccine or drug against CHIKV has been licensed so far^3^. Because of high potential of epidemic and unavailable medical countermeasures, CHIKV used to be considered to be one of the WHO Blueprint priority pathogens^2^. Moreover, CHIKV is classified as BSL3 pathogen and operation of live authentic CHIKV should be conducted in P3 or above containment^4^, which slows down the research progress of CHIKV.

Pseudovirus (PsV) that is an artificially made recombinant viral particle often possesses the similar conformation as authentic live virus and can mimic viral infection. Since the nucleic acid inside the pseudovirus cannot express viral surface membrane proteins, the pseudovirus loses the ability to form a viral particle after the first round of transduction^5^. PsV particles have been widely used as an alternative to the authentic viral particle for drug screening, vaccine evaluation, investigation of pathogenesis, and so on^5,6^. There are two key advantages of PsVs over authentic viruses, particularly those being highly pathogenic. Firstly, PsVs of BSL3 or BSL4 viruses are safer than authentic viruses and can be handled in P2 laboratory. Secondly, reporter genes delivered by PsVs are convenient for the quantification of interactions between viruses and host cells. In fact, the PsV system has been widely used in virology studies, including CHIKV^7^, Lyssaviruses^8^, Middle East respiratory syndrome coronavirus (MERS-COV)^9^, H7N9 influenza viruses^10^, Severe acute respiratory syndrome coronavirus 2 (SARS-CoV-2)^11^ and so on.

CHIKV is a single-stranded, positive-sense RNA virus with a viral genome in 11.5 kb, coding four nonstructural proteins (nsp1-nsp4) and five structural proteins (C-E3-E2-6K-E1). The structural proteins E3-E2-6K-E1 constitutes the envelope of CHIKV. E1 and E2 form a heterodimer that is further assembled into the trimer of spike glycoprotein on the viral surface. The spike protein mediates viral attachment to host cells and facilitates viral infection, and thus, is the major player in the induction of neutralizing antibody^12,13^. E3 mediates the interaction between E1 and E2. The 64 amino-acids glycoprotein 6K participates in the translocation of structural polyproteins and maturation of E2^14,15^. Therefore, these envelope proteins represent critical factors involve in the interaction between virus and host cell. In this study, we firstly constructed a three-plasmid packing system, in which the CHIKV envelope proteins were inset into HIV backbone to form a PsV particle that packed two reporter genes of *ZsGreen1* and *luciferase*. Then we illustrated the structural features of CHIKV PsVs by electron microscopy. Finally, we developed a neutralization assay using these PsV particles and successfully quantified the neutralizing activities of CHIKV-infected human serum samples. Our data indicate that the CHIKV PsV particle offers a powerful alternative system to study authentic CHIKV, making many CHIKV-associated studies doable in a P2 laboratory.

## Results

### Construction of the recombinant plasmid expressing CHIKV envelope proteins

**Figure 1:**
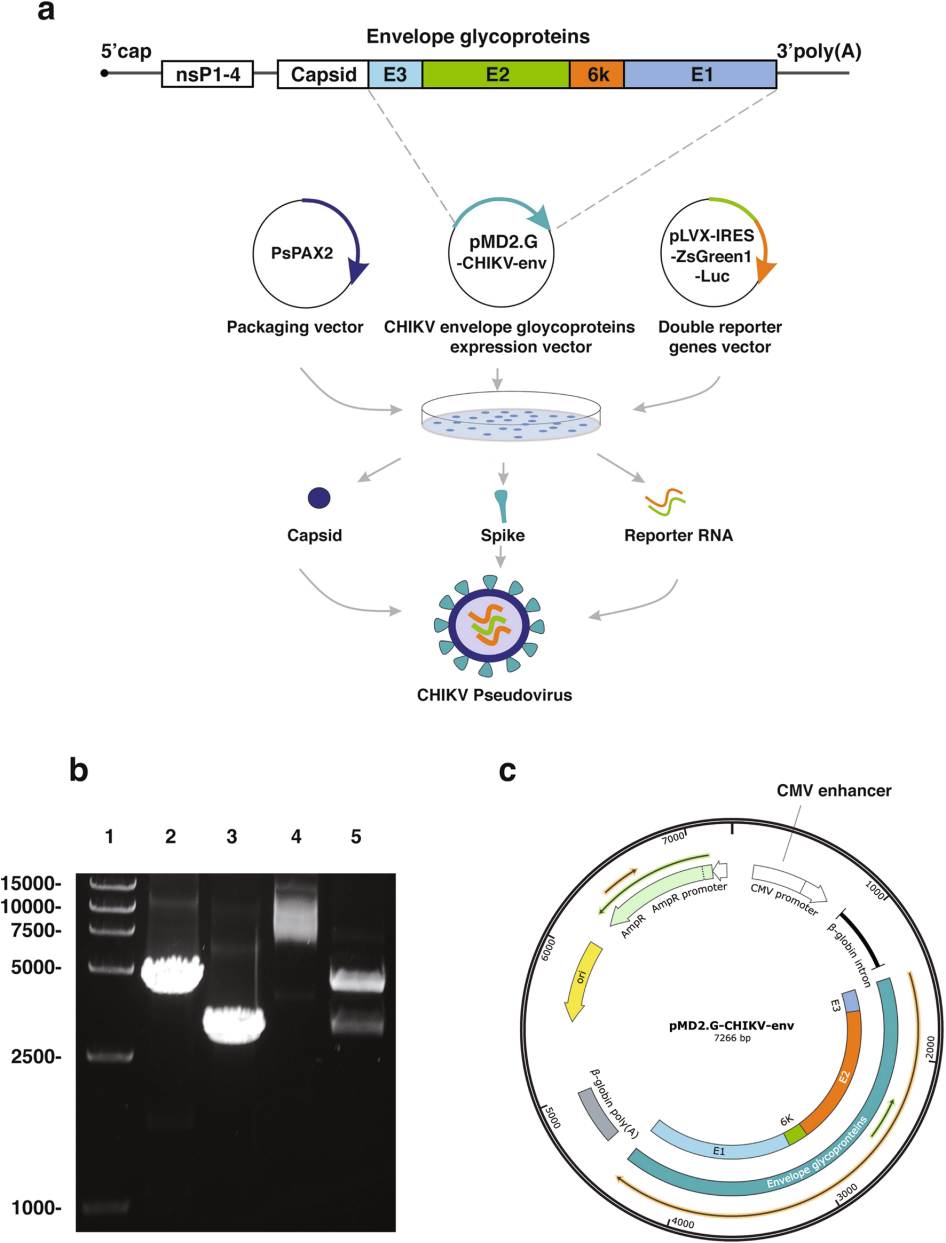
Construction of the CHIKV PsV. (**a**) The CHIKV PsV construction scheme. Each gene fragment was indicated by the colored box. (**b**) Construction and identification of the pMD2.G-CHIKV-env. Primers for PCR were described in the “Materials and methods” section. 5 ul of each product was analyzed in 0.8% agarose gel. Lane 1, DNA marker; Lane 2, linearized pMD2.G-ΔVSV-G backbone (4295 bp); Lane 3, CHIKV envelope gene (2995 bp); Lane 4, undigested pMD2.G-CHIKV-env plasmid (7266 bp); Lane 5, pMD2.G-CHIKV-env plasmid digested by AscI and Xbal (2967 bp and 4299 bp). (**c**) Map of the recombinant plasmid pMD2.G-CHIKV-env that was illustrated by the software SnapGene Viewer (CA, USA).

CHIKV PsV was constructed by HIV-1 lentiviral packaging system (Fig. 1a). Firstly, we performed PCR via specific primers (described in Methods) to obtain the CHIKV envelope gene and the linearized vector pMD2.G-ΔVSV-G. Electrophoresis and sequencing showed that the expected fragments with the sizes of 2995 bp and 4295 bp were successfully amplified (Fig. 1b). Then CHIKV envelope gene was cloned into the linearized pMD2.G-ΔVSV-G vector. The recombinant plasmid was confirmed by restriction digestion analysis (Fig. 1b) and sequencing. These results indicated that the recombinant plasmid expressing CHIKV envelope proteins (pMD2.G-CHIKV-env) was successfully constructed as designated (Fig. 1c).

### Production of CHIKV PsV

**Figure 2:**
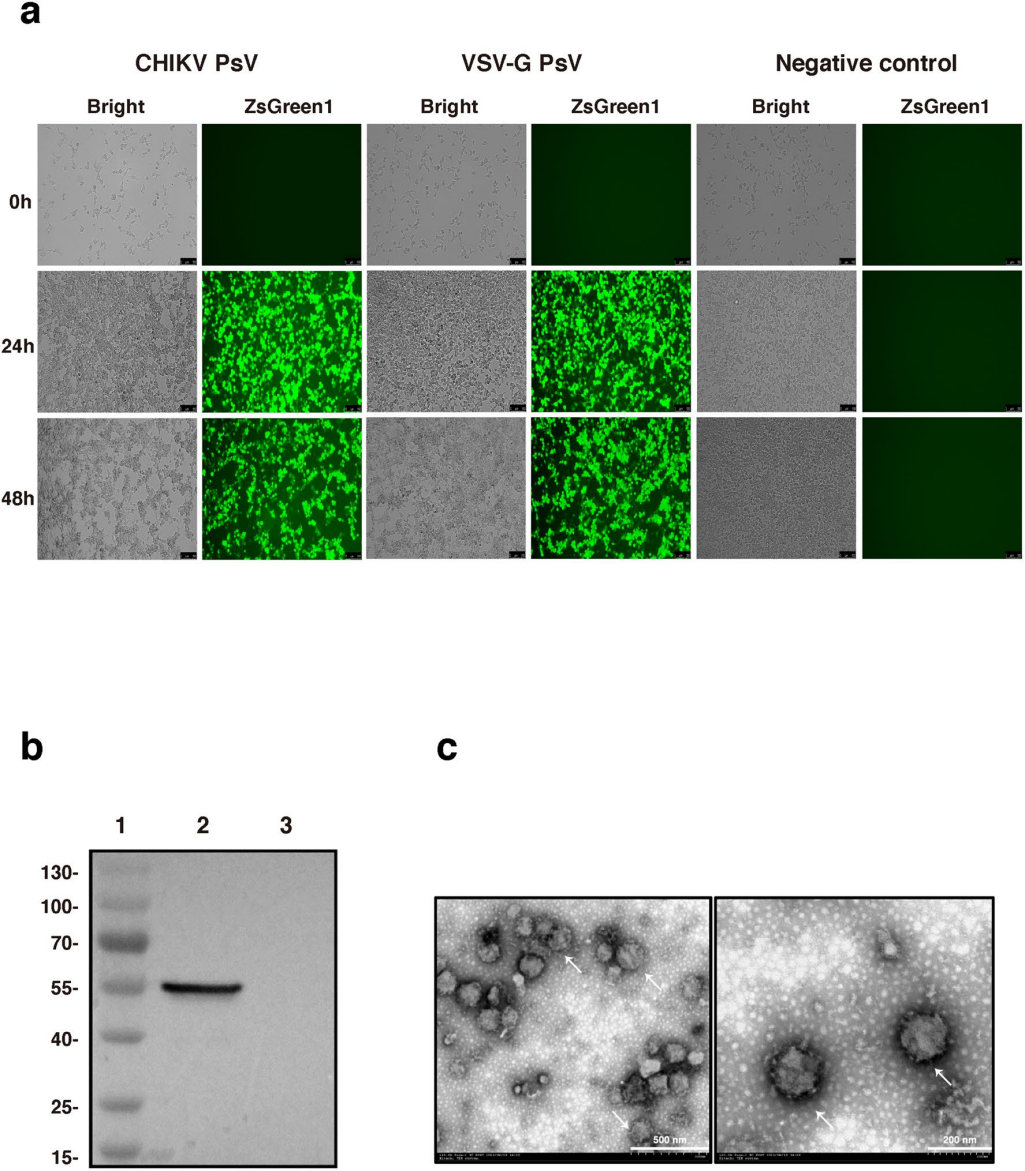
Formation of CHIKV PsV particles. (**a**) Three plasmids (PsPAX2, pLVX-IRES-ZsGreen1-Luc, and pMD2.G-CHIKV-env or pMD2.G) were co-transfected into 293T cells. At the indicated time points, fluorescence was observed under the microscope to monitor the transfection efficiency. (**b**) The PsV particles concentrated by ultracentrifugation were analyzed by Western blotting using mouse Monoclonal antibody against E1. 1: 180 kDa protein marker; 2: the lysate of CHIKV PsVs; 3: the lysate of VSV-G PsVs. Unprocessed images can be found in supplementary information Fig. S1. (**c**) CHIKV PsV particles was stained with phosphotungstic acid and observed under transmission electron microscopy. The scale bars represent 500 nm (left) and 200 nm (right).

The three-plasmid system was used to produce CHIKV PsV, including the CHIKV envelope proteins expressing plasmid pMD2.G-CHIKV-env, the packaging plasmid PsPAX2 and the transfer plasmid pLVX-IRES-ZsGreen1-Luc. At 24 hours post co-transfection, cells were in good conditions and 70% of them expressed the ZsGreen1 fluorescent protein. At 48 hours post co-transfection, there were about 90% of cells expressing fluorescent protein (Fig. 2a), suggesting that these plasmids were efficiently transduced into 293T cells. Furthermore, most fluorescent cells began to shrank, fall off and even die with a grape-cluster-like appearance indicating the release of PsV, which is the possible reason that cells at 24 hpt appeared to be more fluorescent than those at 48 hpt. The results of immunoblotting analysis confirmed the expression of CHIKV envelope proteins in the PsV particles (Fig. 2b). Finally, we observed the formation of CHIKV PsV particles with the size of 80-140 nm and typical enveloped structure under transmission electronic microscope (Fig. 2c). These results suggest that we have successfully produced CHIKV PsV particles.

### Transduction of CHIKV PsV in 293T cells

**Figure 3:**
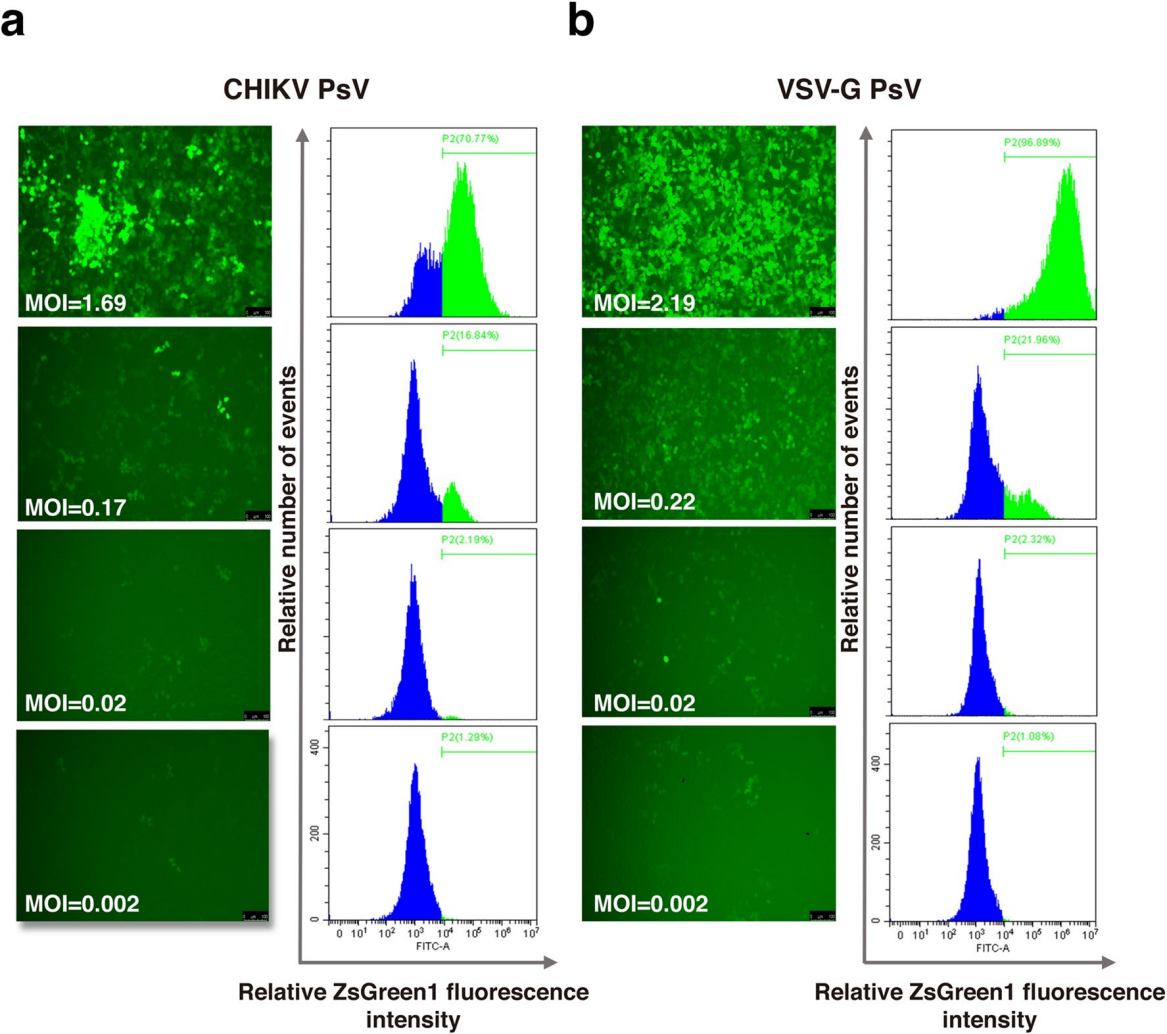
Titration of the CHIKV PsV. 293T cells in the 24-well plate were transduced with serial ten-fold dilutions of PsV particles (**a**) or VSV-G PsV (**b**). At 72 h post transduction, cells were firstly observed under the fluorescence microscope (left panel). Then cells were trypsinized and resuspended for titration via flow cytometric analysis (right panel). Panels in this figure from top to bottom correspond to 1–1000 times dilutions. The MOI was calculated for 293T cells in each well according to the PsV titer. Scale bar: 100 μm.

The transduction of CHIKV PsV was further evaluated in 293T cells. Expression of the reporter ZsGreen1 was monitored under the fluorescence microscope or by flow cytometric analysis. The results showed that CHIKV PsV could transduce and release the reporter ZsGreen1 into 293T cells (Fig. 3a). The titer of CHIKV PsV in this study was 3.16×10^6^ TU/ml, similar to that of the positive control VSV-G PsV (3.29×10^6^ TU/ml) (Fig. 3b).

### Development of CHIKV PsV micro-neutralization assay

**Figure 4:**
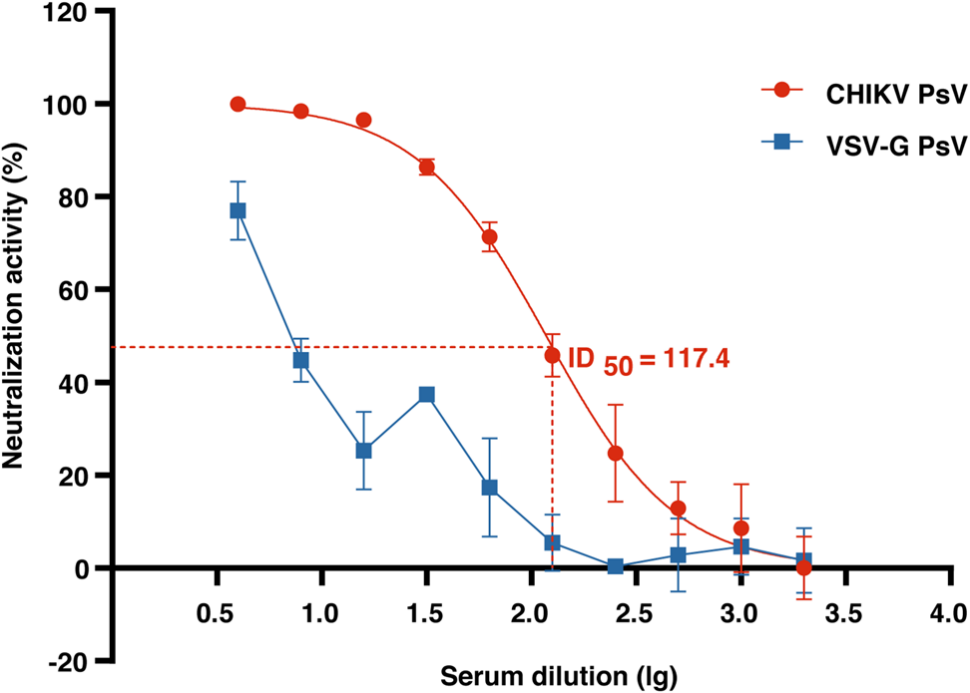
Development of CHIKV PsV micro-neutralization assay. Serially diluted serum from CHIKV-immunized rabbit was incubated with CHIKV or VSV-G PsV. After one-hour incubation, the mixture was transferred to 293T cells. Luciferase activity was measured at 72 h post transduction to evaluate the neutralizing ability of antibody to CHIKV PsV or VSV-G PsV on the microplate reader, followed by the calculation of ID_50_. Error bars show the standard deviations (SD) of results from duplicate independent experiments.

To validate the applicability of the constructed CHIKV PsV in this study, we developed a microneutralization assay using these PsV and serially diluted rabbit serum sample that were known to contain anti-CHIKV antibodies. 500 TU of CHIKV PsVs were used for each neutralization reaction with VSV-G PsV as a control (non-CHIKV envelope proteins control) for the specificity of neutralization. The activity of luciferase was measured on the microplate reader to evaluate the neutralizing ability of antibodies. Compared with the VSV-G PsV, the CHIKV PsV could be specifically neutralized by the antibody in the known CHIKV serum sample. CHIKV PsV transduction was inhibited by the serum sample in a dose-dependent manner. The neutralization titer of serum (50% inhibitory dose, ID_50_) against CHIKV PsV was 117.4 (Fig. 4), indicating that PsV is potentially applicable in the researches of CHIKV.

### CHIKV pseudovirus transduction is inhibited by sera from infected individuals

**Figure 5:**
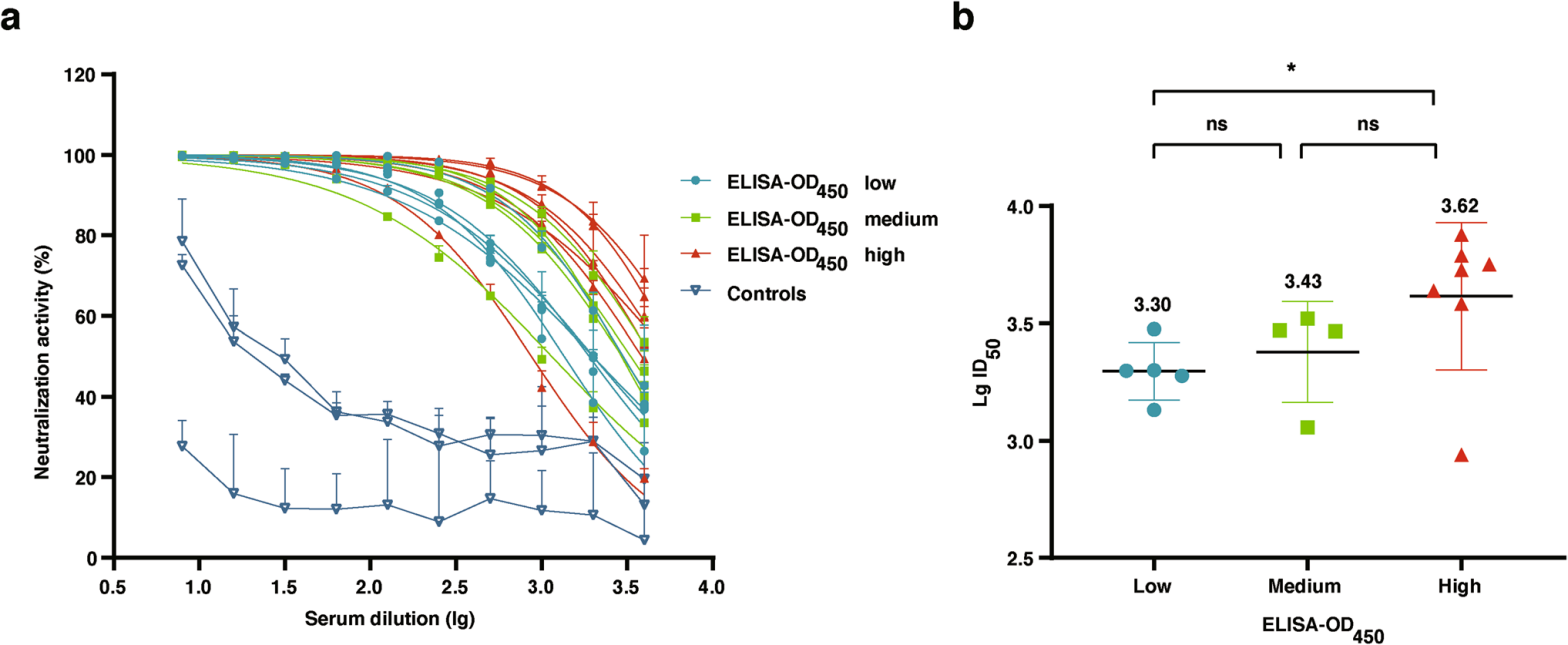
Application of CHIKV PsV micro-neutralization assay in human serum samples. CHIKV PsV neutralization assay was performed with two-fold serial dilutions of CHIKV convalescent human sera. Luciferase activity was measured at 72 h post-transduction of CHIKV PsV. A total of 20 serum samples were used, 17 of them were obtained from CHIKV-positive patients, while 3 serum samples were from CHIKV-negative patients as negative controls. **(a)** The inhibition curves of human serum samples on CHIKV PsV. ID_50_ as neutralizing titer was calculated via the software Prism Graphpad 8.0.2. Error bars show the standard deviations (SD) of results from three independent experiments. **(b)** Correlation between ELISA-OD_450_ groups and CHIKV PsV LgID_50_. The statistical significance analysis was performed by Student’s t test. P values of < 0.05 (^*^) were considered as statistically significant. The mean LgID_50_ is given on the top.

CHIKV convalescent serum samples were firstly analyzed via ELISA. The human serum samples were then divided into three groups according to ELISA-OD_450_ (Table 1). Microneutralization assay was conducted on these samples using CHIKV PsV produced in this study. Through measurement of luciferase activity, most of the CHIKV convalescent human serum samples showed strong neutralization activity in a dose dependent manner (Fig. 5a). Negative control serum samples showed certain neutralization at the first several concentrations, which decreased rapidly and kept at a low level. This result suggested that the neutralization is specifically against CHIKV (Fig. 5a). The CHIKV PsV neutralization titers (PsV NT) were shown in Table 1. In order to analyze the relationship between neutralizing antibody and total IgG antibodies against CHIKV, we made a plot for these convalescent serum samples using the values of ELISA-OD_450_ as x-axis and PsV NTs as y-axis. Through this comparison, we found there was a significantly positive correlation between neutralizing antibody and total IgG antibodies (Fig. 5b).

**Table 1:** Summary of CHIKV convalescent human sera (related to Fig. 5b).

Groups	Experiment number	ELISA-OD_450_	ID_50_ (CHIKV PsV NT)
ELISA-OD_450_ low	H23	0.46	2991
	H24	0.76	1997
	H27	1.00	1988
	L23	1.10	1892
	H37	1.22	1352
ELISA-OD_450_ medium	L1	1.45	4399
	H25	1.62	3308
	H1	1.78	2945
	H7	1.92	2922
	H18	2.11	1141
ELISA-OD_450_ high	L8	2.50	6150
	H58	2.51	3833
	L4	2.65	7548
	H12	2.68	5633
	H29	2.80	5339
	H30	3.03	4346
	L12	3.48	871.2
Controls	1	–	–
	2	–	–
	3	–	–

A total of 20 human serum samples (17 positive samples from CHIKV convalescent individuals, 3 negative samples from healthy individuals were used as controls), were divided into three groups according to ELISA-OD_450_.

## Discussion

Adaptive mutation of A226V facilitates CHIKV to infect *Ae. Albopictus*, contributing to the transmission of mutant CHIKV to broader region than the prototypic viruses^16^. Within last two decades, CHIKV spread rapidly from African continent to Asia, America and Europe, leading to millions of infectious cases and thousands of deaths, which becomes a global concern of public health^16–19^. Therefore, we should make full preparation for this potential outbreak, including elucidation of pathogenesis, drug screening and vaccine development, and so on. However, dependence of CHIKV operation on P3 containment hinders the progress of CHIKV researches due to the limitation of the high-level biosafety facility. In the present study, we constructed CHIKV PsV particles containing CHIKV envelope and two reporters. This system is potentially applicable in studying the interaction between CHIKV and host cell, drug screening, evaluation of vaccine efficacy at the P2 laboratory.

PsV is a kind of virus-like particle with capability of one-time transduction, and made of viral capsid protein and envelope proteins packing non-viral nucleic acid inside. Therefore, PsV is commonly used as a safe alternative for authentic live virus, particularly for highly pathogenic viruses, in studies of viral serology ^20–22^, anti-viral strategy ^13,23,24^, and viral pathogenesis^24–26^. The reporter in PsV is designed for evaluation of the interaction between host cell and PsV. The most common reporter is luciferase because it is very sensitive. However, measurement of luciferase activity is complicate and expensive. Dual reporters (ZsGreen1 and luciferase) have been increasingly engineered into PsV of this study and other reports^27,28^. The fluorescent reporter ZsGreen1 in PsV can be used to monitor efficiency of transfection and titration, which is more convenient and cheaper than the reporter luciferase.

In this study, we constructed PsV particles, where CHIKV envelope was inserted into HIV capsid. Therefore, the formed PsV particle owned the morphology and size of HIV particle (80–140 nm) (Fig. 2c). It is generally believed that the capsid is an essential element for CHIKV to form infectious virus particles^29–32^. Actually, even though capsid is completely deficient in the mutant CHIKV, infectious virus can still be formed although some of morphological characteristics are different from naïve CHIKV^33^.

Prior to the utilization of PsV, it is necessary to determine the titer of viral particle. The copy number of viral genome or concentration of viral protein (such as p21 of HIV-1) is usually used in the titration experiments. However, these methods are not only time-consuming and laborious, but also cannot exclude the non-infectious viral particles, which leads to the higher titer than actual value^4,25,34,35^. In contrast, PsV constructed in this study contained the reporter ZsGreen1. Compared with the traditional titration method on basis of the virus particle itself (qPCR detection of the copy number of viral genome or ELISA measurement of viral protein concentration), ZsGreen1 is expressed only in infected cells, which effectively excludes the interference of non-infectious virus particles, making the titration more accurate. In addition, as a fluorescent signal, ZsGreen1 can be easily captured by flow cytometry, which is easier than the traditional method of titration.

The plaque reduction neutralization test (PRNT) is considered as a gold standard for neutralization assays. However, PRNT needs the use of hazardous live CHIKV and BSL3 facility, which impedes the application of PRNT to CHIKV-related studies. In addition, visualization and counting of plaques in PRNT are time-consuming and laborious procedures. Many studies have demonstrated that the PsV neutralization test could be a good substitute for the plaque reduction test^27,34^. In order to overcome the limitations of PRNT mentioned above, here we developed a PsV neutralization assay. In this assay, luciferase activity was used to quantify the neutralization activity, which had been considered to be semi-automatic, high throughput, sensitive and accurate^27,34,36^. As compared with the neutralization activity of VSV-G PsV, our result showed that neutralization assay of CHIKV PsV is dose dependent and CHIKV specific (Fig. 4). Previous studies have reported that neutralizing antibodies represent a constant proportion of total antibodies^34^. In this study, we found that the ratio of neutralizing antibodies was positively correlated with the total IgG of CHIKV antibodies from infected individuals. This result is consistent with the conclusions of previous studies and further illustrated that CHIKV PsV produced in this study might be a reliable and efficient alternative system for CHIKV research.

In summary, the results of this study demonstrate that CHIKV PsV containing double reporters were successfully produced. These PsV, with one-time transduction of 293T cells, could be potentially used to evaluate the interaction between CHIKV and host cells, which provides a safe and effective method for drug screening and vaccine evaluation in BSL2 laboratories.

## Materials and methods

### Reagents

The plasmids PsPAX2 (Cat#HG-VMA0649), pLVX-IRES-ZsGreen1-Luc (Cat#HG-VMH1035), and pMD2.G (Cat#HG-VMA0648) were purchased from Honorgene Company (China, Changsha). The competent Stable cells (Cat#BC118-01) were obtained from AngYuBio Inc. The FUGENE HD Transfection Reagent (Cat#E2311) and Bright-Glo™ Luciferase Assay System (Cat#E2620) were purchased from Promega (USA), and mouse anti-CHIKV-E1 monoclonal antibody from R&D (USA, Cat#MAB97792), HRP-conjugated anti-mouse IgG from Proteintech (USA, Cat#SA00001-1).

### Construction of the plasmid pMD2.G-CHIKV-env

The gene coding CHIKV envelope proteins (Asia lineage strain 1151D4f; GenBank accession no. OK316992) was amplified via RT-PCR with primers that contained two enzyme sites AscI and Xbal, CHIKV Env-F 5′-TTGGCGCGCCAAGCCACCATGAGTCTGGCCATTCCAGTTAT-3′ and CHIKV Env-R 5′-GCTCTAGAGCTTAGTGCCTGCTAAACGACACG-3′. To add two enzyme sites AscI and Xbal to the vector pMD2.G, PCR was used to amplify the backbone pMD2.G-ΔVSV-G with primers, pMD2.G-ΔVSV-G-F 5′-CTAGTCTAGACTAGCCTGCACAACAGATTCTTCATGT-3′ and pMD2.G-ΔVSV-G-R 5′-TTGGCGCGCCAAACAGATCGATCTCTGTTGAATTCAG-3′. These two fragments amplified by PCR were digested with two enzyme sites AscI and XbaI, followed by ligation via T4 ligase and transformation into the competent Stable cells. The recombinant plasmid, named as pMD2.G-CHIKV-env, was confirmed by specific enzymes and sequencing.

### Preparation of CHIKV PsV

Three-plasmid system was used to construct CHIKV PsV, in which the HIV-based lentivirus packaging vector PsPAX2, which contains lentiviral Gag/Pol, was used to provide the capsid for PsV. And the plasmid pLVX-IRES-ZsGreen1-Luc that contained two reporter genes, *luciferase* and *ZsGreen1*, was used to provide RNA genome for PsV. The third plasmid pMD2.G-CHIKV-env containing CHIKV envelope fragment, was used to provide spike glycoprotein for PsV. A schematic diagram of the construction of CHIKV PsV is shown in Fig. 1a.

These three plasmids were co-transfected into 293T cells (kindly provided by Dr. Longding Liu, the Institute of Medical Biology, China) using the FuGENE HD Transfection Reagent. The VSV-G PsV system that consists of three plasmids, PsPAX2, pLVX-IRES-ZsGreen1-Luc and pMD2.G for vesicular stomatitis virus (VSV-G) spike glycoprotein, was used as a positive control for transfection and production of PsV. The reporter ZsGreen1 was use to evaluate the transfection efficiency. The culture supernatant was harvested at 48 h post transfection, followed by centrifugation at 2000 *g* for 5 min. After passing through 0.45 μm membrane, the supernatant PsV particles was aliquoted and stored at − 80 °C for further analysis.

### Identification of CHIKV PsV

The filtered supernatant containing PsV was added to the top of 20% sucrose solution in the ultracentrifuge tube, followed by ultracentrifugation at 25,000 rpm for 2.5 h with the setting of acceleration 9 and deceleration 3 (Beckman Coulter, Optima MAX-XP)^37–39^. After the supernatant was discarded, 1% volume of DMEM was used to resuspend the pellet of PsV particles that was then kept at 4 °C overnight. Next day, the suspension of PsV particles was ready for further analysis. In western blotting, mouse anti-CHIKV E1 mAb (0.5 μg/ml) and HRP-conjugated anti-mouse IgG antibody were used to detect CHIKV envelope protein. Transmission electron microscopy (TEM, HITACHI H-7650) was utilized to confirm the formation of PsV particles. Ten microliter of filtered supernatant was added to the Copper mesh, followed by absorption for 2 min at room temperature. Excessive water in the Copper mesh was then removed. Phosphotungstic acid counterstaining was conducted on samples. After removal of excessive water, stained samples were kept in a dish for 30 min and observed under TEM.

### Titration of PsV particle (flow cytometry)

The titer of the PsV was determined by transduction of HEK 293T with serial ten-fold dilutions of PsV particles. 0.5–1 × 10^5^ cells were seeded per well in a 24-well plate (500 μl)^37^. In each well, 500 μl of serially diluted PsV was added in the presence of 8 μg/ml polybrene. After 72 h of incubation, the percentage of ZsGreen1 positive cells was determined by flow cytometry (Beckman, Cytoflex, USA). The highest dilution of PsV at which ZsGreen1 positive cells percentage was below 40% was used to calculate the titer as follows, Transduction Units (TU/ml) = (percentage of fluorescent positive cells) × (cell number per well on the day of transduction) × (PsV dilution factor). On basis of the titer of PsV and number of cells seeded in each well, the multiplicity of infection (MOI) here can be calculated as follows, Multiplicity of Infection (MOI) = the volume of CHIKV PsV × the titer of CHIKV PsV/ the number of cells.

### ELISA for the antibody IgG against CHIKV

The commercial kit CHIKjj *Detect* IgG ELISA (Ref# CHKG-C, WA USA) was used to measure the antibody IgG against CHIKV in human serum samples. ELISA was performed according to the procedures recommended by the manufacture *InBios*. The serum sample was diluted 1/100 in the Sample Dilution Buffer provided. The readout of this assay is the optical density at 450 nm in a microplate reader.

### Micro-neutralization assay

Neutralization assay was performed in the 96-well plate as described previously^40^. The CHIKV PsV were diluted to 1×10^4^ TU/ml and 50 µl of them were mixed with a 2-fold serial dilution (Fig. 4 and Fig. 5a) of serum samples from CHIKV-immunized rabbits or CHIKV convalescent patients (kindly provided by Yunnan Provincial Key Laboratory of Vector-borne Diseases Control and Research, Yunnan Institute of Parasitic Diseases). The mixture was incubated at 37 ℃ for 1 to 2 h and then added to the cells (50,000/well) in each well. After 72 h of incubation, the expression of the reporter ZsGreen1 was observed under the fluorescence microscope (Leica, DMIL LED) and the activity of luciferase was measured via Bright-Glo™ Luciferase Assay System according to the protocol recommended by the manufacture. The neutralization curve was made on basis of the activity of luciferase. And neutralization titer of serum samples against CHIKV PsV was expressed as the 50% inhibitory dose (ID_50_) that was calculated via the inhibition curve.

### Statistical data analysis

Determination of ID_50_ values were performed using the GraphPad Prism 8.0.2 software (La Jolla, CA, USA) as nonlinear regression, which is expressed as log(inhibitor) vs. normalized response–Variable slope. The statistical significance analysis was performed by Student’s *t* test. *p* values of<0.05 (*) were considered as statistical significance.

## Supplementary Information

Supplementary Figure S1.
