# Supplementary material for: Preparation and application of chikungunya pseudovirus containing double reporter genes
Source: Sci Rep. 2022 Jun 14;12:9844. doi: 10.1038/s41598-022-13230-0 (PMC9194775; doi:10.1038/s41598-022-13230-0)
Supplement: Supplementary file 1 — Supplementary Figure S1. [file 41598_2022_13230_MOESM1_ESM.docx]

**Supplementary information**

**Manuscript:**

**Preparation and application of Chikungunya pseudovirus containing double reporter genes**

Chunyan Su^1^, Kaiyun Ding^1^, Jingwen Xu^1^, Jianchao Wu^1^, Jiansheng Liu^1^, Jiayuan Shen^2^, Hongning Zhou^2^, Hongqi Liu^1,^ **^*^**

^1^Institute of Medical Biology, Chinese Academy of Medical Sciences and Peking Union Medical College, Kunming, 650118, China; ^2^Yunnan Provincial Key Laboratory of Vector-borne Diseases Control and Research, Yunnan Institute of Parasitic Diseases, Simao Pu’ er, Yunnan 665000, China


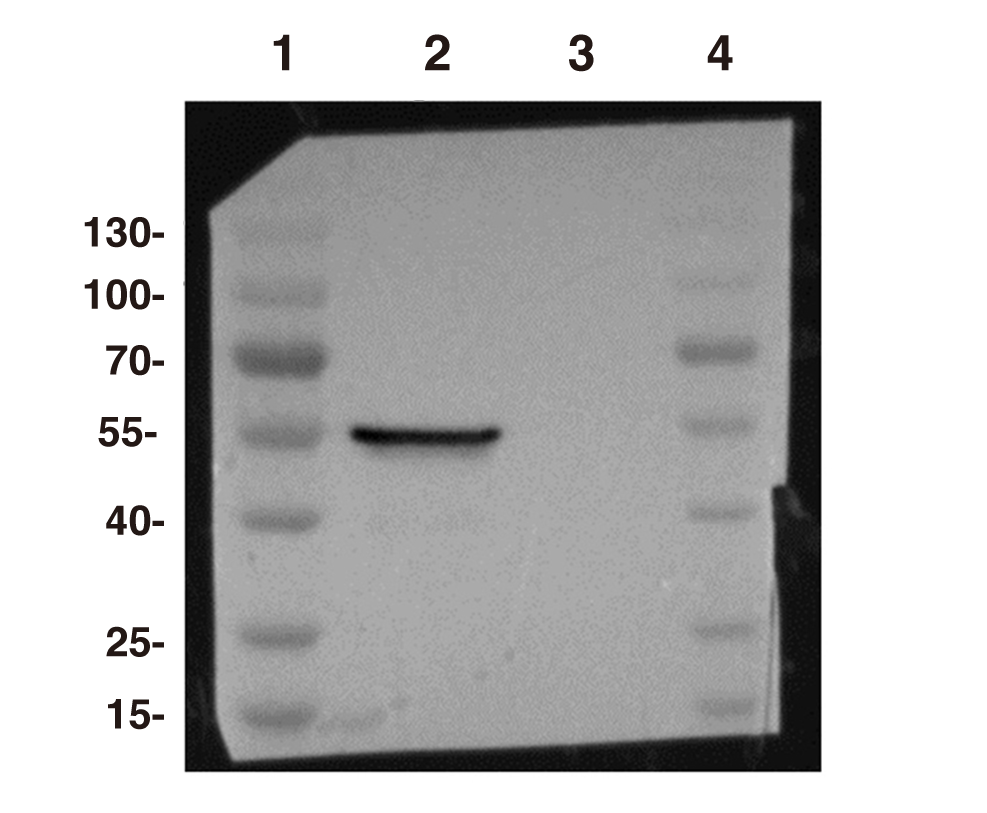


**Figure S1. Production of CHIKV pseudovirus (PsV).** The PsV particles concentrated by ultracentrifugation were analyzed by Western blotting using mouse Monoclonal antibody against E1. 1: 180 kDa protein marker; 2: the lysate of CHIKV PsVs; 3: the lysate of VSV-G PsVs.
